# Feasibility of combined screening for upper gastrointestinal adenocarcinoma risk by serology and Cytosponge testing: the SUGAR study

**DOI:** 10.1136/jclinpath-2019-205700

**Published:** 2019-06-24

**Authors:** Yiwang Xu, Ahmad Miremadi, Alexander Link, Peter Malfertheiner, Rebecca C Fitzgerald, Jan Bornschein

**Affiliations:** 1 MRC Cancer Unit, Hutchison/MRC Research Centre, University of Cambridge, Cambridge, UK; 2 Histopathology Department, Cambridge University Hospitals NHS Trust, Cambridge, UK; 3 Dept. of Gastroenterology, Hepatology and Infectious Diseases, Otto-von-Guericke-University, Magdeburg, Germany; 4 Translational Gastroenterology Unit, John Radcliffe Hospital, Oxford University Hospitals, Oxford, UK

**Keywords:** pepsinogens, TFF3, barrett’s oesophagus, gastric atrophy, gastric cancer

## Abstract

**Aims:**

Aim was to assess the feasibility of serum markers to identify individuals at risk for gastro-oesophageal adenocarcinoma to reduce the number of individuals requiring invasive assessment by endoscopy.

**Methods:**

Blood samples from 56 patients with Barrett’s oesophagus and 202 non-Barrett controls who previously took part in a trial assessing the accuracy of the Cytosponge for Barrett’s oesophagus were assessed for serum pepsinogen (PG) 1 and 2, gastrin-17, trefoil factor 3 (TFF3) and *Helicobacter pylori* infection.

**Results:**

PG1 was pathological (<50 ng/mL) in 26 patients (10.1%), none of whom had Barrett’s oesophagus (p<0.001). Smoking and drinking had no influence on these results. Pathological PG1 was associated with stomach pain (p=0.029), disruption of sleep (p=0.027) and disruption of diet by symptoms (p=0.019). Serum TFF3 was not associated with any clinical parameter.

**Conclusions:**

Assessment of serum PG1 could be combined with a test for Barrett’s oesophagus to identify additional patients requiring endoscopy.

## Introduction

Epithelial metaplasia is a premalignant condition in the upper gastrointestinal tract. This includes Barrett’s oesophagus, the precursor for oesophageal adenocarcinoma and intestinal metaplasia of the stomach for gastric cancer. National and international evidence-based guidelines recommend endoscopic surveillance of Barrett’s oesophagus, and European consensus guidelines recommended a similar approach for advanced preneoplastic conditions of the stomach in 2012.^1^

Oesophagogastroduodenoscopy and biopsy sampling coupled with histopathological review remains the gold standard for diagnosis of precancerous conditions. However, for most parts of the Western world population-based endoscopic screening is neither feasible nor cost-effective due to the low prevalence of Barrett’s and gastric premalignant conditions. Thus, prescreening of the general population with minimally or non-invasive tests to identify individuals at higher risk in whom further endoscopic assessment should be undertaken is desirable. Serum pepsinogens (PGs) are an accepted surrogate marker for glandular atrophy of the gastric body.^2 3^ The diagnostic properties of this test are appropriate for its application in both Asian and Western populations.^4–6^ Additional assessment of gastrin-17 (G17) in the serum could add further information.^7^

In some parts of Asia, combined assessment of serum PGs as markers for gastric mucosal integrity and anti-*Helicobacter pylori* antibodies is already established for population-based screening. Group stratification has shown that while individuals with positive *H. pylori* status are at increased risk of gastric cancer development, those with pathological serum PG (usually varying in the literature between 30 and 70 ng/µL) indicating gastric mucosal atrophy carry an at least sixfold further increased risk.^8 9^

A serum-based test has not yet been identified to aid in the diagnosis of Barrett’s oesophagus, but the minimally invasive Cytosponge has demonstrated promising accuracy and acceptability for the detection of Barrett’s as a triage test for endoscopy.^10^ The device samples cells from the gastric cardia and along the length of the oesophagus. The key marker for immunohistopathological assessment of mucosal fragments acquired by the Cytosponge is trefoil factor 3 (TFF3) which identifies intestinal metaplasia.^11^ The Cytosponge does not sample the mid and distal portions of the stomach, and therefore, complementary approaches are required to identify individuals at risk for gastric cancer. TFF3 has also been reported to be a promising serum marker for preneoplastic changes of the stomach.^12 13^

This study aims to assess the feasibility of combined serological assessment of PG1, PG2, G17, TFF3 and anti-*H. pylori* antibodies in a cohort that has been tested with the Cytosponge to identify additional patients who might benefit from endoscopic investigation.

Blood samples were collected in standard citrate serum tubes as part of the Barrett's Oesophagus Screening Trial 2 (BEST2) before ingestion of the Cytosponge and endoscopy.^10^ Samples were immediately spun down and frozen at −80°C. Written informed consent was obtained from all subjects prior to sampling and any intervention. A cohort of n=273 patients was selected randomly to be assessed for *H. pylori* IgG, PG1, PG2 and G17 in the serum with a combined ELISA kit (GastroPanel, Biohit Healthcare, Finland), as well as a TFF3 ELISA-based serum test (Human TFF3 Quantikine ELISA kit, R&D Systems, Abingdon, UK).

The cohort comprised ‘control’ patients with upper GI symptoms but without a diagnosis of Barrett's oesophagus or other previously known upper gastrointestinal pathology (n=202), patients with Barrett's oesophagus (n=56), including 38 patients with non-dysplastic Barrett’s oesophagus (NDBE) and 18 patients with high-grade dysplasia or intramucosal cancer (HGD/IMC). Due to the known problems with interobserver agreement, patients with low grade dysplasia or indefinite for dysplasia were excluded from the analysis (n=15). The serology results were correlated with the Cytosponge-test results, the endoscopic findings and the available clinical data (table 1).

**Table 1:** Demographic and serological data

		Controls (n=202)	NDBE (n=38)	HGD/IMC (n=18)	Total* (N=258)	P value
Sex	Male, (%)	77 (38.1)	31 (81.6)	15 (83.3)	123 (47.7)	**<** **0.001**
Age	Years, median (range)	55.5 (20–91)	67.0 (35–88)	65.5 (50–79)	58.0 (20–91)	**<** **0.001**
*Helicobacter pylori*	Positive (%)	34 (16.8)	3 (7.9)	3 (16.7)	40 (15.5)	0.409
Barrett’s length	cm, median (range)	0 (0–0.5)	4 (1–16)	5 (1–14)	0 (0–15)	**<** **0.001**
BMI	kg/m^2^, median (range)	26.4 (16.5–45.0)	28.3 (21.7–43.9)	28.4 (22.9–36.4)	26.8 (16.5–45.0)	**0.008**
Smoker	Positive (%)	26 (12.9)	3 (7.9)	3 (16.7)	32 (12.4)	0.570
Heavy drinker	Positive (%)	19 (9.4)	8 (21.1)	2 (11.1)	29 (11.2)	0.091
PPI use	Positive (%)	125 (61.9)	33 (86.6)	17 (94.4)	175 (67.8)	**<** **0.001**
PG1	ng/mL, median (range)	100.7 (6.05–500)	286.8 (58.4–500)	312.2 (132.3–500)	124.2 (6.1–500)	**<** **0.001**
PG2	ng/mL, median (range)	6.0 (0.8–60)	12.8 (3.2–25.7)	17.5 (7.7–60)	7.24 (0.8–60)	**<** **0.001**
G17	ng/mL, median (range)	4.8 (0–50)	12.7 (0–50)	15.2 (0–50)	6.5 (0.0–50.0)	**0.004**
TFF3	ng/mL, median (range)	7.3 (0.6–31.3	7.5 (2.1–11.7)	7.3 (4.1–12.2)	7.3 (0.6–31.3)	0.979
PG1 <50 ng/mL	Positive (%)	26 (12.9)	0 (0.0)	0 (0.0)	26 (10.1)	**0.011**
G17 >10 ng/mL	Positive (%)	75 (37.1)	20 (52.6)	12 (66.7)	107 (41.5)	**0.017**
TFF3 >7 pg/mL	Positive. (%)	101 (54.3)	20 (52.6)	9 (50.0)	130 (53.7)	0.921

*Patients with Barrett’s oesophagus indefinite for dysplasia have been excluded from this part of the analysis. P-values are printed in bold if statistically significant in group comparison.

BMI, body mass index; G17, gastrin-17; HGD/IMC, high-grade dysplasia or intramucosal cancer; NDBE, non-dysplastic Barrett’s oesophagus; PG, pepsinogen; PPI, proton pump inhibitor; TFF3, trefoil factor 3.

In addition to the assessment of absolute serum values, cut-off values were chosen according to the current literature to divide data into pathologically abnormal and normal results (pathological PG1 <50 ng/mL; PG1/2-ratio <3.0; G17 >10 ng/mL). There is no literature on generally accepted cut-offs for serum TFF3.

Non-parametric tests were applied for comparison of continuous variables, and Fisher's exact test for comparison of categorical variables. Statistical analyses were performed using SPSS V.22.0 (IBM). Significance was assumed for a two-sided p<0.05.

## Results

**Figure 1:**
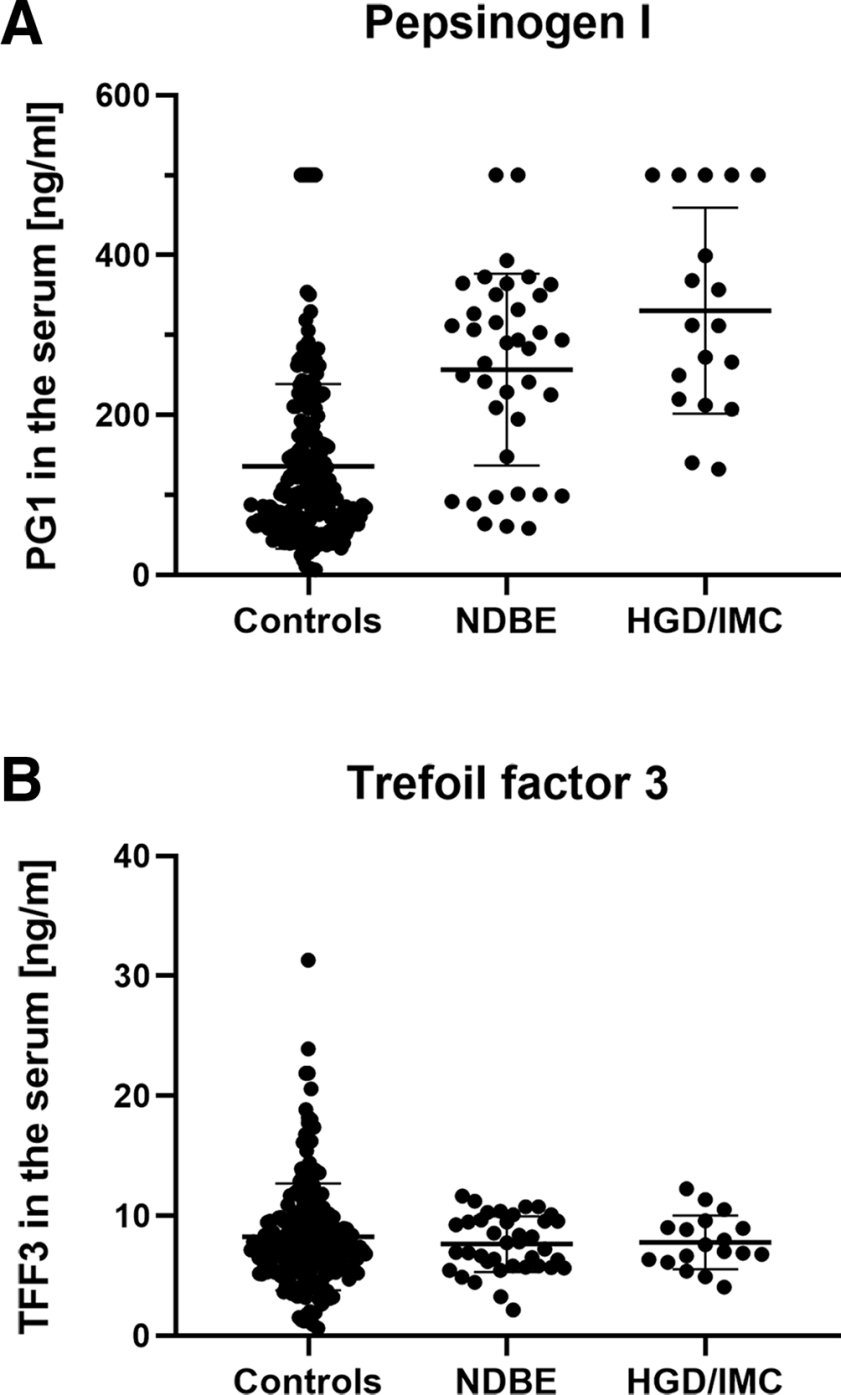
Comparison of serum values for PG1 (A) and TFF3 (B). Displayed is the distribution of serum values for PG1 (A) and TFF3 (B) for individuals with non-dysplastic Barrett’s oesophagus (NDBE), more advanced oesophageal lesions, including high-grade dysplasia and intramucosal cancer (HGD/IMC) and non-Barrett’s ‘controls’. Group comparison was done by Kruskal-Wallis test with significance being assumed for p<0.05.

Patients without a diagnosis of Barrett's oesophagus had significantly lower serum values for PG1 (100.7 vs 292.0 ng/mL), PG2 (6.0 vs 13.9 ng/mL) and G17 (4.8 vs 13.4 ng/mL) than those with Barrett's metaplasia (p<0.001). Interestingly, mean absolute values for PG1 were higher in HGD/IMC (312.2 ng/mL) compared with patients with NDBE (286.8 ng/mL vs controls: 100.7 ng/mL) (p<0.001; figure 1A). With reference to the literature, serum PG1 <50 ng/mL was considered pathological as was a PG1/2 ratio <3.0 and a serum G17 >10 ng/mL. Pathological results for PG1 were present in 26 (10.1%) and for the PG1/2-ratio in only 2 (0.8%) patients. None of the patients with Barrett’s oesophagus showed pathological PG1 results compared with 12.9% of the individuals without Barrett’s oesophagus (p<0.001; table 1). A pathological G17 value (ie, high G17 serum values) was also more often seen in patients without Barrett’s oesophagus (57.1% vs 37.1%, p=0.009). PG1 (r=0.433, p<0.001), PG2 (r=0.441, p<0.001), and G17 (r=0.182, p=0.003) in the serum showed a positive association with the maximum length of the Barrett's segment. A pathological PG1/2-ratio was only found in two control patients, so this was not analysed further.

There was no statistically significant difference in the serum levels for TFF3 between the patient groups (p=0.979; figure 1B). In addition, there was no correlation for the serum values of TFF3 with the length of Barrett’s oesophagus.

**Figure 2:**
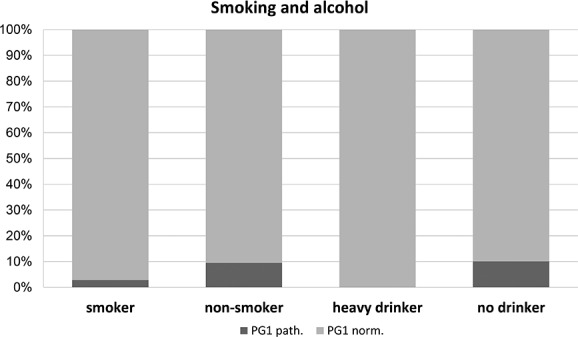
Proportion of patients with pathological PG1 test in relation to smoking and drinking habits. Displayed is the proportion of patients with pathological PG1 test (<50 ng/L) in patients depending on the smoking and drinking habits. Indicated are current smokers as well as patients who considered themselves as heavy drinkers (ie, regular consumption of alcohol above the recommended limit). PG1 test results were not different between smokers and non-smokers (p=0.753), and there was a trend for a higher proportion for positive PG1 test results in patient who were not regular drinkers (p=0.54). PG1, pepsinogen I.

**Figure 3:**
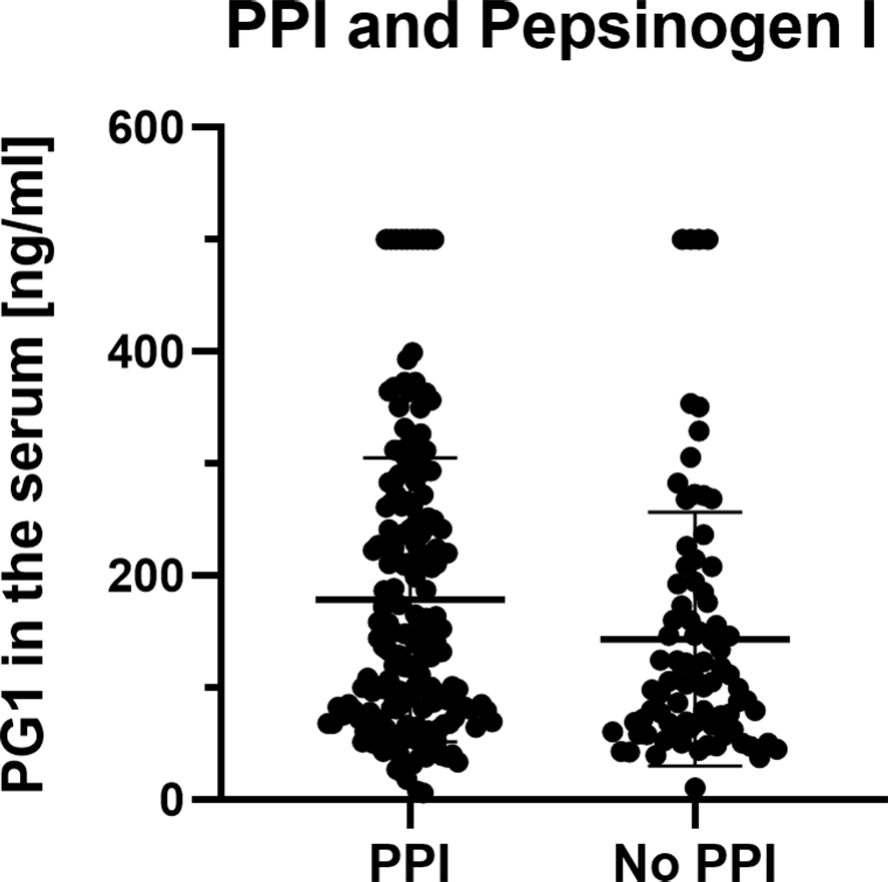
Comparison of serum PG1 in patient with or without regular PPI intake. Displayed is the distribution of serum values for PG1 for individuals with and without regular PPI intake. Group comparison was done by Mann-Whitney U test with significance being assumed for p<0.05. PPI, proton pump inhibitor.

Risk factors such as smoking and drinking habits had no significant influence on the serum parameters (figure 2). There was, however, a trend for patients with alcohol consumption above the UK recommended limit to show fewer pathological PG1 results compared with individuals who did not drink heavily (0.0% vs 10.1%, p=0.054). Active smokers showed a broadly similar proportion of individuals with a pathological PG1 test compared with non-smokers (6.3% vs 10.8%; p=0.753). Proton pump inhibitors (PPIs) intake was associated with higher PG1 (140.5 ng/mL vs 105.0 ng/mL; p=0.026; figure 3) and PG2 values, but not with elevated serum levels of TFF3. As expected, the diagnosis of Barrett’s oesophagus was associated with an increased use of PPI. Of patients with Barrett's oesophagus 89.3% had a PPI prescription whereas only 61.9% of patients without Barrett's oesophagus took regularly PPI (p<0.001). TFF3 serum results did not show any association with the parameters mentioned above.

**Figure 4:**
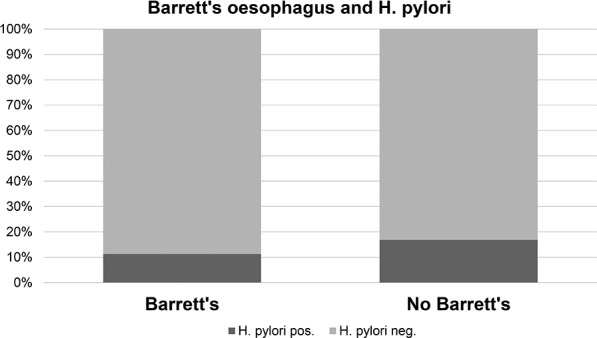
Association of Barrett’s oesophagus and *Helicobacter pylori* infection. There was no statistically significant difference in the serological *H. pylori* status in patients with or without diagnosis of Barrett’s oesophagus (p=0.304; Fisher’s exact test).

Only 40 patients (15.0%) were positive for *H. pylori* infection by serology and rapid urease test on biopsy, which is lower than in the general population in the UK. The previously reported inverse association between positive *H. pylori* status and the diagnosis of Barrett's oesophagus could not be confirmed in our cohort, but our study was not powered for this analysis. There was no statistical difference in the *H. pylori* prevalence between patients with or without Barrett’s oesophagus (16.8% vs 10.7%; p=0.304; figure 4).

## Discussion

This study aimed to assess the feasibility of combined screening for upper gastrointestinal adenocarcinoma risk in patients with dyspeptic or reflux-related symptoms. All individuals had undergone minimally invasive assessment for Barrett’s oesophagus with the Cytosponge.^10^ It is of note that patients with Barrett’s oesophagus did not show pathologically altered serum PGs suggesting that they are more likely to have an intact gastric mucosa. This is in line with previous studies suggesting a mutually exclusive relationship whereby either reflux-related changes or gastric preneoplastic changes occur in patients with adenocarcinoma at the gastro-oesophageal junction.^14^ This and the correlation of the serum level of PG1 with the length of the Barrett’s segment support the hypothesis that an intact gastric mucosa and the related acid output contributes to neoplastic processes in the distal oesophagus under reflux conditions.

The generally low prevalence of 15.5% *H. pylori* positive patients and pathological PG1 levels in 10.1% of patients is comparable to a cohort study from the Netherlands, which enrolled patients undergoing screening colonoscopy and demonstrated a positive *H. pylori* status in 22% and preneoplastic conditions of the stomach in 9.3% of their cohort.^15^ Interestingly, the previously reported inverse association between *H. pylori* infection and Barrett’s oesophagus could not be confirmed in our study which was, however, not powered to do so.

The diagnostic properties of PG testing are adequate for population-based prescreening to identify individuals who would require further assessment by endoscopy,^2 4 5^ allowing prediction of the individual risk of developing gastric cancer.^8 9 16^ Assessment of serum PGs in our cohort identified extra 26 patients in whom further diagnostic assessment by endoscopy would be appropriate, in addition to individuals who have tested positive for Barrett’s oesophagus by the Cytosponge TFF3 test). With the broad majority of studies on PG testing having been published on Asian cohorts, it is important also to compare our results with data from Western cohorts. Prevalence of gastric pathology showed a range from 3.4% to 10.8% in studies from several European countries.^3 6 17 18^ A study from Portugal with a particularly low number of positive test results concluded that there would be one gastric cancer per 2200 serum tests, or one case per 74 positive tests.^18^

Interestingly, assessment of the PG1/2-ratio, which has been reported to be more robust than PG1 alone, does not yield further information in our cohort. This could be due to the fact that increased PG2 in the serum (resulting in a decreased PG1/2-ratio) is primarily due to a higher degree of mucosal inflammation, which is controlled by PPI treatment in our study (85% PPI users). In addition to this, PPI users generally show a higher PG1 level in the serum which leads to further imbalance in the PG1/2-ratio. PG1 on its own is a good indicator for pathology in the gastric body with elevated levels usually being associated with higher progression rates.^19^ Further confounding factors like smoking or heavy drinking had, on the other hand, only minor effects on the serum test results without reaching statistical significance.

There is ongoing debate whether assessment of G17 adds any substantial value to PG assessment. Our data suggest that this is indeed not the case and this marker could probably be omitted. G17 is a highly unstable peptide and requires strictly standardised sampling conditions, making its use in routine clinical practice difficult. Although biologically a highly relevant factor due to its oncogenic potential, results of G17 need to be interpreted with great caution.

Although serum TFF3 has been reported as a stable marker for gastric atrophy, assessment of TFF3 did not add further value to that of PG1. We tested stratification according to previously published cut-off values without identifying any benefit.^12 13^ TFF3 in the serum was associated with a positive TFF3 immunohistochemistry signal in the Cytosponge samples, but there was no correlation with the length of Barrett’s mucosa. This indicates that serum TFF3 might be a discriminative marker indicating presence of IM in the distal oesophagus, rather than a quantitative one allowing conclusions on the length of the metaplastic segment. TFF3 is abundantly expressed in the intestinal epithelium, and therefore, serum values might be influenced by various factors and are probably not reliable diagnostic markers. However, further studies focussing on the diagnostic applicability and the appropriate cut-off thresholds need to be undertaken to investigate the clinical applicability of this marker.

This study is clearly limited by the fact that no standard gastric biopsies were obtained on the study cohort to confirm gastric pathology. The diagnostic properties of serum PG testing have, however, been extensively studied in the past, and we considered that this information was sufficient for this pilot study.

The identification of patients with (advanced) preneoplastic conditions of the stomach and subsequent endoscopic surveillance of these mucosal changes remains currently the best option for gastric cancer prevention and early detection.^1 20^ Prospective validation is needed to assess if the combination of a serum markers for gastric pathology with the minimally invasive Cytosponge test for Barrett’s oesophagus could be a potentially complimentary strategy to identify patients at risk for upper gastrointestinal adenocarcinoma.
